# Inhibition of platelet aggregation and thrombosis by indole alkaloids isolated from the edible insect *Protaetia brevitarsis seulensis* (Kolbe)

**DOI:** 10.1111/jcmm.13055

**Published:** 2016-12-20

**Authors:** JungIn Lee, Wonhwa Lee, Mi‐Ae Kim, Jae Sam Hwang, MinKyun Na, Jong‐Sup Bae

**Affiliations:** ^1^ College of PharmacyChungnam National UniversityDaejeonRepublic of Korea; ^2^ College of PharmacyCMRIResearch Institute of Pharmaceutical SciencesBK21 Plus KNU Multi‐Omics based Creative Drug Research TeamKyungpook National UniversityDaeguRepublic of Korea; ^3^ Department of Agricultural BiologyThe National Academy of Agricultural Science, RDAWanju‐gunRepublic of Korea

**Keywords:** *Protaetia brevitarsis seulensis* (Kolbe), indole alkaloids, coagulation cascade, fibrinolysis

## Abstract

*Protaetia brevitarsis seulensis* (Kolbe) has been temporarily registered as a food material by the Ministry of Food and Drug Safety of Korea (MFDS). The current study aimed to discover small antithrombotic molecules from this edible insect. Five indole alkaloids, 5‐hydroxyindolin‐2‐one (**1**), (1*R*,3*S*)‐1‐methyl‐1,2,3,4‐tetrahydro‐β‐carboline‐3‐carboxylic acid (**2**), (1*S*,3*S*)‐1‐methyl‐1,2,3,4‐tetrahydro‐β‐carboline‐3‐carboxylic acid (**3**), (3*S*)‐1,2,3,4‐tetrahydro‐β‐carboline‐3‐carboxylic acid (**4**) and L‐tryptophan (**5**), were isolated from the insect. Among them, compounds **1** and **2** prolonged aPTT and PT and impaired thrombin and FXa generation on HUVEC surface. Moreover, these compounds inhibited platelet aggregation. Antithrombotic effects of compounds **1** and **2** were further confirmed in pre‐clinical models of pulmonary embolism and arterial thrombosis. Collectively, these results demonstrated that compounds **1** and **2** could be effective antithrombotic agents and serve as new scaffolds for the development of antithrombotic drug.

## Introduction

The leading causes of death worldwide are diseases that involve the heart and blood vessels and, consequently, thrombosis 1. Most thromboembolic processes require anticoagulant therapy. This explains the current effort to develop specific and potent anticoagulant and antithrombotic agents 1. Thrombus formation due to an abnormal coagulation process is often observed in arteries or veins and may result in reduced blood flow or ischaemia 1. Platelet activation in atherosclerotic arteries is central to the development of arterial thrombosis; therefore, a precise control of platelet function is imperative in preventing thrombotic events 2. Because of the central role of platelets in cardiovascular thrombosis, current and investigational antiplatelet therapies target key pathways of platelet activation 3. These targets include platelet surface receptors (*e.g*. P2Y purinoceptor 12 (P2Y12), integrin αIIbβ3, protease activated receptor (PAR)‐1, glycoprotein 1b, P‐selectin and the thromboxane prostanoid receptor), signalling molecules (*e.g*. cyclooxygenase 1 (COX1)) and endothelial products (nitric oxide) 3. Therefore, antiplatelet therapy is a well‐established part of the treatment of cardiovascular disease including the COX 1 inhibitor aspirin, the P2Y12 antagonist (clopidogrel, ticagrelor or prasugrel) and integrin αIIbβ3 antagonists 3. However, limitations of current therapies include weak inhibition of platelet function (by aspirin), blockade of only one pathway of ADP‐mediated signalling (by clopidogrel), slow onset of action (of clopidogrel), interpatient response variability with poor inhibition of platelet response in some patients (to clopidogrel), the inability to transform the success of intravenous integrin αIIbβ3 antagonist therapy into oral therapy and the inability to completely separate a reduction in thrombotic events from an increase in bleeding events 3, 4, 5, 6. The insufficient antithrombus and antiplatelet effect of the present armamentarium might explain the vascular relapses. Most thromboembolic processes require anticoagulant therapy. This explains the current effort to develop specific and potent anticoagulant and antithrombotic agents. Research on novel bioactive compounds and drugs with different mechanisms of action, increased efficacy, safety and pharmacokinetics, and low toxicity is highly needed 1.

Insects have been considered as potential food and drug resources. In a recent study, we revealed the anticoagulant activity of small‐molecule alkaloids from *Scolopendra subspinipes mutilans*, lactams which have been applied for cardiovascular disorders 7. Bioactive compounds including new norepinephrine derivatives, sesquiterpenoids and lactams were also discovered from the edible insect *Aspongopus chinensis* Dallas and were effective against pain, dyspepsia and kidney diseases 8. The venom derived from samsum ant (*Pachycondyla sennaarensis*) was known to have antitumour and anti‐inflammatory effects, and could improve the immune system 9. In Korea, mealworms, the larvae of *Tenebrio molitor* that are used to treat liver disease and dementia have been approved as a food material by the MFDS 10. The larvae of *Protaetia brevitarsis seulensis* (Kolbe) have also been temporarily approved by the MFDS as a food material since September 2014 11. *P. brevitarsis seulensis*, belonging to the Cetoniidae family, is a white‐spotted flower chafer that is widely distributed in Korea, China, Japan, Taiwan and Europe 12. The insect has been used as a functional food and traditional medicine to treat breast cancer, inflammatory disease, hepatic cancer, liver cirrhosis and hepatitis 11; particularly, peptides from its larvae have been shown to possess antibacterial activity 13. A composition analysis of the larvae revealed the presence of fatty acids, volatile constituents and nutrient substances 14. Its fatty acids have been shown to induce apoptosis mediated by caspase‐3 activation in tumour cells 15. Up to date, there are limited studies characterizing the small‐molecule metabolites of *P. brevitarsis seulensis*. In our preliminary study, the whole body extract of the larvae extended blood coagulation time with no toxicity in human umbilical vein endothelial cells (HUVECs). This study thus aimed to discover the antithrombotic compounds in the insect, which resulted in the isolation of five indole alkaloids. The indole alkaloids **1‐5** were tested for their antithrombotic activity by monitoring the prothrombin time (PT), activated partial thromboplastin time (aPTT) and platelet aggregation. Furthermore, we examined the antithrombotic effect using the ferric chloride (FeCl_3_)‐induced thrombosis animal models.

## Materials and methods

### General experimental procedures

IR data were recorded on a Thermo Electron US/Nicolet 380 (Madison, WI, USA). Nuclear Magnetic Resonance (NMR) experiments were carried out using a Bruker Avance III (600 MHz) spectrometer. HRESIMS data were obtained on a JMS 700 high‐resolution mass spectrometer (JEOL, Tokyo, Japan). Vacuum liquid chromatography (VLC) was conducted on Merck silica gel (70–230 mesh), and medium‐pressure liquid chromatography (MPLC) was carried out utilizing a Biotage Isolera apparatus equipped with a reversed‐phase C18 SNAP Cartridge KPC18‐HS (340 g; Biotage AB, Uppsala, Sweden). Preparative reversed‐phase HPLC (prep HPLC) separation was carried out on an YMC C_18_ column (250 × 20.0 mm, 5 μm) or on a Kinetex Biphenyl column (250 × 21.5 mm, 5 μm) at 5 ml/min. flow rate.

### Extraction and isolation

Freeze‐dried whole body of *Protaetia brevitarsis seulensis* (Kolbe) (5.17 kg) was refluxed three times with 1% acetic acid in ethanol (5 L × 4). The ethanolic extract was evaporated under reduced pressure to yield a brownish ethanol extract of 968.0 g. The extract was suspended in water and partitioned successively with *n*‐hexane, ethyl acetate and butanol to yield an *n*‐hexane‐soluble fraction (400 g, PBS‐1), an ethyl acetate soluble fraction (10.0 g, PBS‐2), a butanol‐soluble fraction (47.3 g, PBS‐3) and a residue (510.0 g, PBS‐4). The solvent fractions were tested for their effects on PT, aPTT in human plasma. Of these, the ethyl acetate‐ and the butanol‐soluble fractions displayed anticoagulant activity. The ethyl acetate fraction (PBS‐2) was subjected to VLC, eluting with a stepwise gradient of hexane/ethyl acetate 5:1, 4:1, 2:1 and 1:1; hexane/ethyl acetate/methanol, 1/1/0.2; chloroform/methanol, 7:1; chloroform/methanol 5:1, followed by washing with methanol as eluent to yield 7 fractions (PBS‐2a‐g). A part of the PBS‐2d (2 g) fraction eluting with hexane/ethyl acetate/methanol 1:1:0.2 was purified by preparative HPLC on a Kinetex Biphenyl column using a gradient of methanol in water from 30% to 70% to obtain 5‐hydroxyindolin‐2‐one (*t*_R_, 22.0 min., 12 mg) (**1**). The butanol‐soluble fraction (47.8 g, PBS‐3) was fractionated into 6 fractions (PBS‐3a‐f) by VLC, eluting with a stepwise gradient of chloroform/methanol, 9:1, 7:1, 6:1, 4:1 and 2:1. The PBS‐3f fraction (20 g) was further separated by MPLC eluting with methanol in water from 20% to 50%, followed by washing with methanol as eluent to yield 3 fractions (PBS‐3f‐a‐c). Four alkaloids, (1*S*,3*S*)‐1‐methyl‐1,2,3,4‐tetrahydro‐β‐carboline‐3‐carboxylic acid (*t*_R_, 58 min., 15 mg) (**2**), (1*R*,3*S*)‐1‐methyl‐1,2,3,4‐tetrahydro‐β‐carboline‐3‐carboxylic acid (*t*_R_, 57 min., 20 mg) (**3**), (3*S*)‐1,2,3,4‐tetrahydro‐β‐carboline‐3‐carboxylic acid (*t*_R_, 53 min., 25 mg) (**4**) and L‐tryptophan (*t*_R_, 35 min., 1 g mg) (**5**) were isolated from the PBS‐3f‐b (2 g) fraction by preparative HPLC on a Kinetex Biphenyl column using a gradient of methanol in water from 10% to 40%. The purity (>95%) of each isolated compound was checked by HPLC‐UV (210 nm).

### Cell culture

Primary HUVECs were obtained from Cambrex Bio Science (Charles City, IA, USA) and were maintained as described previously 16. Briefly, cells were cultured in EBM‐2 basal media supplemented with growth supplements (Cambrex Bio Science, Charles City, IA, USA) at 37°C under 5% CO_2_ atmosphere until confluent. All experiments were performed with HUVECs at passage 3–5.

### *In vitro* and *ex vivo* platelet aggregation assay

The *in vitro* platelet aggregation study was performed according to a previously reported method 17, 18. Platelet‐rich plasma (PRP) was incubated with the indicated concentration of each compound in DMSO for 1, 3, 5 or 10 min. They were subsequently stimulated by U46619 (2 μM) in 0.9% saline solution or collagen (1 μg/ml) at 37°C for 5 min. Platelet aggregation was recorded using an aggregometer (Chronolog, Havertown, PA, USA). For the *ex vivo* aggregation assay, male mice were fasted overnight and the indicated concentration of each compound in DMSO was administered by intravenous (i.v.) injection. After 24 hrs, PRP (10^9^ platelets/ml) in a volume of 240 μl was incubated at 37°C for 1.5 min. in the aggregometer under continuous stirring at 1000 r.p.m. and subsequently stimulated with U46619 (2 μM). Platelet aggregation was recorded as described above.

### Coagulation assay

The aPTT and PT were determined using a Thrombotimer (Behnk Elektronik, Norderstedt, Germany) as per the manufacturer's instructions and as described previously 19. Briefly, citrated normal human plasma (90 μl) was mixed with 10 μl of heparin or of each compound and was incubated for 1 min. at 37°C. Subsequently, the aPTT assay reagent (100 μl) was added and the plasma sample was incubated for an additional 1 min. at 37°C, followed by the addition of 20 mM CaCl_2_ (100 μl). The clotting times were recorded. For the PT assays, citrated normal human plasma (90 μl) was mixed with 10 μl of each compound's stock solution and was incubated for 1 min. at 37°C. The PT assay reagent (200 μl), which had been pre‐incubated for 10 min. at 37°C, was subsequently added and the clotting time was recorded. The PT results were expressed in seconds and as international normalized ratios (INR): INR = (PT sample/PT control)^ISI^, where ISI = international sensitivity index. The aPTT results were expressed in seconds. All experimental protocols (KNUH 2012‐01‐010) were approved by the Institutional Review Board of Kyungpook National University Hospitals (Daegu, Republic of Korea).

### *In vivo* bleeding time

Tail bleeding times were measured using the method described by Dejana *et al*. 19. Briefly, C57BL/6 mice were fasted overnight prior to the experiments. One hour after the i.v. administration of each compound, the tails of the mice were transected at 2 mm from their tips. The bleeding time was defined as the time elapsed until the bleeding stopped. Bleeding times exceeding 15 min. were recorded as lasting for 15 min.

### *Ex vivo* clotting time

Male C57BL/6 mice were fasted overnight and each compound in 0.2% dimethyl sulfoxide (DMSO) was administered by i.v. injection. One hour after the administration, arterial blood samples (0.1 ml) were collected in 3.8% sodium citrate (1/10, v/v) for the *ex vivo* aPTT and PT determination. The clotting times were determined as described above.

### Thrombin activity assay

Each compound in 50 mM Tris–HCl buffer (pH 7.4) containing 7.5 mM EDTA and 150 mM NaCl was mixed. Following a 2‐min incubation at 37°C, thrombin solution (150 μl, 10 U/ml) was added, followed by incubation at 37°C for 1 min. S‐2238 (a thrombin substrate. 150 μl, 1.5 mM) solution was subsequently added and the absorbance at 405 nm was monitored for 120 sec. using a spectrophotometer (TECAN, Männedorf, Switzerland).

### Factor Xa activity assay

The FXa assay was performed using the same method as described for the thrombin activity assay, except for the use of FXa (1 U/ml) and S‐2222 as substrates instead. *Please see Data S1 for more methods.

## Results

### Isolation and structural determination of small‐molecule alkaloids from *P. brevitarsis seulensis*

Chemical investigation of the EtOH extract of *P. brevitarsis seulensis* larvae resulted in the isolation of a series of indole alkaloids **1**‐**5** (Fig. S1). The structures of the isolated compounds were determined by MS, 1D and 2D NMR analysis.

Compound **1** was isolated as a white powder. The ^1^H NMR spectrum showed signals for three aromatic protons with an ABX spin system at δ_H_ 6.78 (1 H, d, *J* = 2.1 Hz), 6.70 (1 H, d, *J* = 8.3 Hz), 6.65 (1 H, dd, *J* = 8.3, 2.1 Hz) and one methylene proton at δ_H_ 3.37 (2 H, s). Inspection of the ^13^C NMR spectra revealed six aromatic carbons at δ_C_ 153.5, 136.9, 127.9, 114.4, 113.4, 110.3, one carbonyl carbon at δ_C_ 176.5 and methylene carbon at δ_C_ 36.5. The HMBC correlations between δ_H_ 3.37 (H‐3) and δ_C_ 176.5 (C‐2)/136.9 (C‐7a) helped to determine the structure as 5‐hydroxyindolin‐2‐one (Fig. S2).

Compound **2** was isolated as a brown powder, [α]$\overset{18}{D}$ ‐112 (*c* 0.2, H_2_O). The ^1^H NMR spectrum showed typical signals of a tryptophan derivative, where four aromatic protons at δ_H_ 7.62 (1 H, d, *J* = 7.7 Hz), 7.47 (1 H, d, *J* = 7.7 Hz), 7.27 (1 H, *t*,* J* = 7.7 Hz) and 7.18 (1 H, *t*,* J* = 7.7 Hz), one methylene at δ_H_ 3.40 (1 H, dd, *J* = 16.4, 5.5 Hz) and 3.12 (1 H, ddd, *J* = 16.4, 9.2, 0.9 Hz), two methine at δ_H_ 4.99 (1H, dd, *J* = 13.7, 6.8 Hz) and 4.26 (1H, dd, *J* = 9.2, 5.5 Hz), and one methyl group at δ_H_ 1.69 (3H, d, *J* = 6.9 Hz) were observed. The ^1^H and ^13^C NMR data analysis revealed the structure to be 1‐methyl‐1,2,3,4‐tetrahydro‐β‐carboline‐3‐carboxylic acid. The absolute configuration of the C‐1 and C‐3 positions was determined to be 1*R* and 3S on the basis of the coupling constants (*J*_H‐1/H‐2_ 13.7, 6.8 Hz and *J*_H‐3 and H‐2/H‐4_ 9.2, 5.5 Hz) 20.

Compound **3** was isolated as a brown powder, [α]$\overset{18}{D}$‐17 (*c* 0.2, H_2_O). The NMR spectroscopic data of compound **3** were similar to that of **2**. The stereochemistry of **3** was assigned as 1*S* and 3*S* based on the coupling constant (*J*_H‐1/H‐2_ 6.2 Hz and *J*_H‐3 and H‐2/H‐4_ 11.9, 4.4 Hz) 20. Thus, the compound **3** was identified as (1*S*,3*S*)‐1‐methyl‐1,2,3,4‐tetrahydro‐β‐carboline‐3‐carboxylic acid 20.

Compound **4** was isolated as a brown powder, [α]$\overset{18}{D}$‐17 (*c* 0.5, MeOH). Different from compounds **2** and **3** by a methyl group at C‐1, compound **4** was identified as (3*S*)‐1,2,3,4‐tetrahydro‐β‐carboline‐3‐carboxylic acid 20.

### Effect of the isolated compounds on platelet aggregation

**Figure 1:**
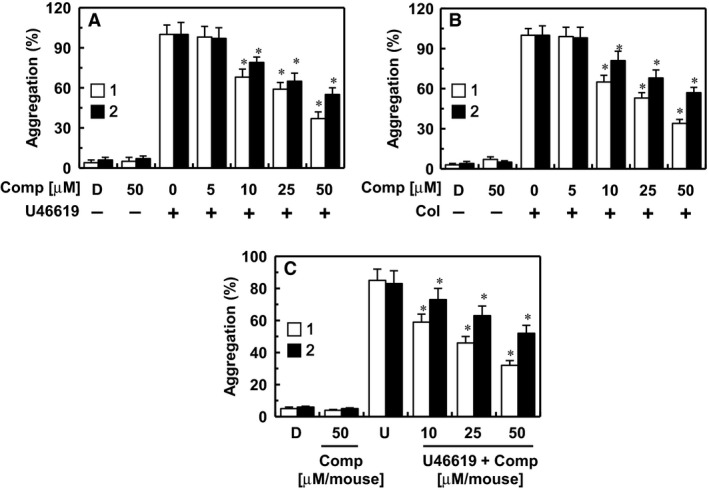
Effects of compounds 1 and 2 on platelet aggregation. (**A**,** B**) The effect of each compound on human platelet aggregation induced by 2 μM U46619 (**A**) or 1 μg/ml collagen (**B**). (**C**) Each compound in DMSO was injected intravenously at the indicated concentration. The effects of each compound on mouse platelet aggregation induced by 2 μM U46619 (U) were monitored *ex vivo*. *D* = 0.2% DMSO used as the vehicle control. *P < 0.05 *versus* U46619 (**A**,** C**) or collagen (**B**) alone.

The effect of each compound on a U46619 (a stable thromboxane A2 analogue/aggregation agonist) or collagen‐induced platelet aggregation was performed as described in the ‘Materials and methods’ section. As shown in Figure 1A and B, treatment with compounds **1** and **2** significantly inhibited human platelet aggregation induced by U46619 (final concentration: 2 μM) or collagen (final concentration: 1 μg/ml) in a concentration‐dependent manner. These *in vitro* results were confirmed in an *ex vivo* platelet aggregation assay (i.v. injection, Fig. 1C). The average circulating blood volume for mice is 72 ml/kg 21. Because the average weight of the mouse used in this study was 27 g and the average blood volume is 2 ml, the amount of compound **1** (2.9, 7.5, or 14.9 μg per mouse) and **2** (4.6, 11.5, or 23.0 μg per mouse) equalled a peripheral blood concentration of approximately 10, 25 or 50 μM, respectively.

### Effects of the isolated compounds on protein kinase C activation and intracellular Ca^2+^ mobilization

**Figure 2:**
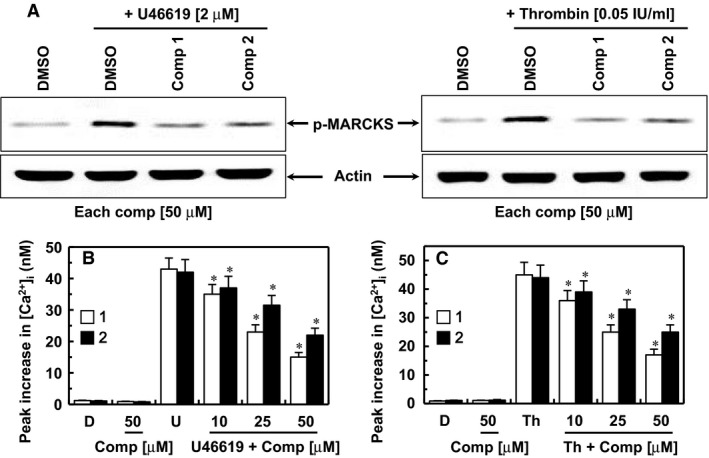
Effects of compounds 1 and 2 on PKC activation and intracellular calcium mobilization. (**A**) Platelet‐rich plasma (PRP) was incubated with DMSO or each compound (50 μM) at 37°C for 10 min. and was stimulated with U46619 (2 μM, left) or thrombin (0.05 U/ml, right) for another 1 min. Phospho‐MARCKS in the platelet lysates was detected using Western blotting (cropped images from full‐length gels). (**B**,** C**) Fura‐2‐loaded human platelets were incubated with DMSO (**D**), compound **1** (white box) and compound **2** (black box) at 37°C for 10 min. in the presence of 1 mM extracellular Ca^2+^, followed by the addition of U46619 (B, 2 μM) or thrombin (**C**, 0.05 U/ml) to trigger the increase in [Ca^2+^]_i_. *D* = 0.2% DMSO used as the vehicle control. The data represent the means ± S.E.M. of three independent experiments performed in triplicate. *P < 0.05 *versus* U46619 (**B**) or Th (**C**) alone.

We next investigated the selectivity of each compound for the signalling pathways that regulate platelet aggregation. Upon platelet stimulation by agonists, phospholipase C (PLC) hydrolyses phosphatidylinositol 4,5‐bisphosphate to diacylglycerol and inositol‐1,4,5‐triphosphate, which promote the activation of protein kinase C (PKC) and increase cytosolic Ca^2+^, respectively 22. PKC and Ca^2+^ act synergistically to induce the granular secretion and activation of glycoprotein (GP) IIb/IIIa, the receptor responsible for the final step of platelet aggregation 22. In this study, the effect of each compound on PKC activation was determined by measuring the phosphorylation of MARCKS, which is a major substrate of PKC in human platelets 23. At the concentrations required to prevent platelet aggregation, compounds **1** and **2** inhibited U46619 (left)‐ and thrombin‐induced (right) MARCKS phosphorylation (Fig. 2A). The changes in intracellular [Ca^2+^]_i_ were measured in U46619‐ and thrombin‐stimulated, fura‐2‐loaded platelets by monitoring the fluorescence of fura‐2. Compounds **1** and **2** inhibited the U46619 (Fig. 2B)‐ and thrombin‐induced increases (Fig. 2C) in [Ca^2+^]_i_.

### Effect of the isolated compounds on the clotting time

Incubation of human plasma with compounds **1** and **2** affected coagulation. The anticoagulant activity of each compound was evaluated using aPTT and PT assays and human plasma (Table 1). As shown in Table 1, the aPTT and PT were significantly prolonged by each compound. Furthermore, the anticoagulant activity of compound **1** was higher than that of heparin or low molecular weight heparin (LMWH). However, (1*S*,3*S*)‐1‐methyl‐1,2,3,4‐tetrahydro‐β‐carboline‐3‐carboxylic acid (**3**), (3*S*)‐1,2,3,4‐tetrahydro‐β‐carboline‐3‐carboxylic acid (**4**) and L‐tryptophan (**5**) did not alter the *in vitro* coagulation time (Table S3). Compounds **1** and **2** at 24.61 and 35.86 μM, respectively, doubled the clotting time in the aPTT assay and at concentrations of 17.06 and 46.46 μM, respectively, doubled the clotting time in the PT assay. Therefore, our results indicate that compounds **1** and **2** can inhibit the blood coagulation pathway.

**Table 1:** Anticoagulant activity of compounds 1 and 2 from *P. brevitarsis seulensis* a

*In vitro* coagulant assay				
Sample	Dose	aPTT (s)	PT (s)	PT (INR)
Control	DMSO	23.7 ± 0.4	11.7 ± 0.4	1.00
Comp 1	5 μM	23.8 ± 0.2	12.0 ± 0.3	1.06
	10 μM	42.3 ± 0.4^b^	14.9 ± 0.5^b^	1.79^b^
	25 μM	54.4 ± 0.5^b^	27.7 ± 0.6^b^	7.91^b^
	50 μM	64.8 ± 0.4^b^	54.9 ± 0.4^b^	40.37^b^
Comp 2	5 μM	23.3 ± 0.4	11.9 ± 0.4	1.04
	10 μM	38.7 ± 0.3^b^	13.7 ± 0.6^b^	1.46^b^
	25 μM	46.2 ± 0.7^b^	15.4 ± 0.4^b^	1.93^b^
	50 μM	52.4 ± 0.5^b^	25.4 ± 0.5^b^	6.43^b^
LMWH	10 IU/ml	31.5 ± 0.6^b^	12.2 ± 0.4	1.11
Heparin	0.5 mg/ml	57.8 ± 0.4^b^	26.7 ± 0.6^b^	7.24^b^

*In vivo* bleeding time (i.v. injection)			
Sample	Dose	Tail bleeding time (s)	*n*
Control	DMSO	30.5 ± 1.2	5
Comp 1	7.5 μg/mouse	52.3 ± 1.0^b^	5
	14.9 μg/mouse	69.6 ± 1.4^b^	5
Comp 2	11.5 μg/mouse	41.2 ± 1.4^b^	5
	23.0 μg/mouse	51.6 ± 1.2^b^	5
LMWH	20 IU/mouse	35.2 ± 0.8^b^	5
Heparin	1 mg/mouse	61.2 ± 1.0^b^	5

a Each value represents the means ± S.E.M. (*n* = 5).

b *P* < 0.05 as compared to control.

### Effects of the isolated compounds on thrombin and FXa activity

**Figure 3:**
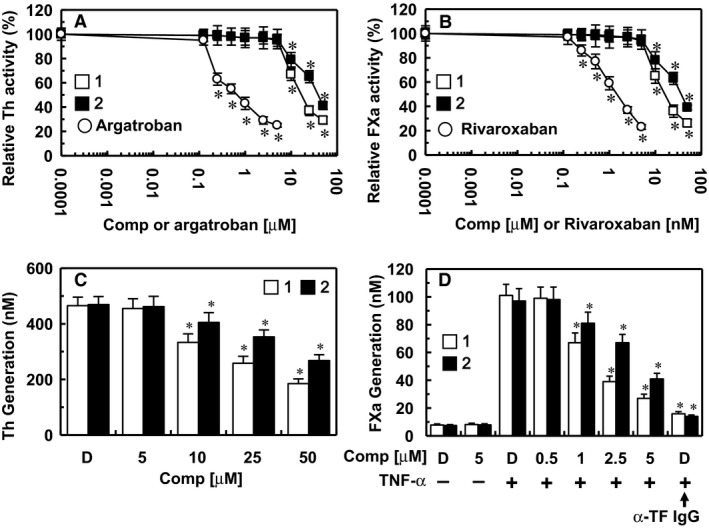
Effects of compounds 1 and 2 on the inactivation and production of thrombin and factor Xa. (**A**) Inhibition of thrombin (Th) by compound **1** (white box) and compound **2** (black box) was measured using a chromogenic assay, as described in the ‘Materials and methods’ section. (**B**) The inhibition of factor Xa (FXa) by each compound was also monitored using a chromogenic assay, as described in the ‘Materials and methods’ section. Argatroban (**A**) or rivaroxaban (**B**) was used as a positive control. (**C**) The HUVEC monolayer was pre‐incubated with FVa (100 pM) and FXa (1 nM) for 10 min. and then with the indicated concentrations of each compound. Prothrombin was added at a final concentration of 1 μM and prothrombin activation was determined after 30 min., as described in the ‘Materials and methods’ section. (**D**) HUVECs were pre‐incubated with the indicated concentrations of each compound for 10 min. TNF‐α (10 ng/ml for 6 hrs)‐stimulated HUVECs were incubated with FVIIa (10 nM) and FX (175 nM) in the absence or presence of anti‐TF IgG (25 μg/ml); the FXa production was determined as described in the ‘Materials and methods’ section. *D* = 0.2% DMSO used as the vehicle control. *P < 0.05 *versus* 0.0001 μM each compound, or argatroban (**A**) or 0.0001 μM each compound, or 0.0001 nM rivaroxaban (**B**), DMSO (**C**) or TNF‐α alone (**D**).

To elucidate the mechanism responsible for the inhibition of coagulation by compounds **1** and **2**, the inhibition of thrombin and FXa activity was determined using chromogenic substrates. Treatment with compounds **1** and **2** resulted in a dose‐dependent inhibition of the amidolytic activity of thrombin, indicating a direct inhibition of thrombin activity by compounds **1** and **2** (Fig. 3A). The direct thrombin inhibitor, argatroban, was used as a positive control. In addition, compounds **1** and **2** inhibited the activity of FXa (Fig. 3B). The direct FXa inhibitor, rivaroxaban, was used as a positive control. These results are consistent with those of our antithrombin assay and therefore suggest that the antithrombotic mechanism underlying the compound **1** and **2**'s actions involves the inhibition of the blood coagulation pathway.

### Effects of the isolated compounds on thrombin and FXa production on HUVECs

In a previous study, Sugo *et al*. reported that endothelial cells support prothrombin activation by FXa 24. In the current study, pre‐incubation of HUVECs with FVa and FXa in the presence of CaCl_2_ before the addition of prothrombin resulted in thrombin production (Fig. 3C). In addition, treatment with compounds **1** and **2** caused a dose‐dependent inhibition of prothrombin‐produced thrombin (Fig. 3C). According to findings reported by Rao *et al*., the endothelium provides the functional equivalent of pro‐coagulant phospholipids and supports the activation of FX 25. Furthermore, in TNF‐α‐stimulated HUVECs, the activation of FX by FVIIa was dependent on TF expression 26. Thus, we investigated the effects of compounds **1** and **2** on the activation of FX by FVIIa. HUVECs were stimulated with TNF‐α to induce TF expression, causing a 13‐fold increase in the rate of FX activation by FVIIa in the stimulated HUVECs (101.5 ± 7.5 nM) compared with that in the non‐stimulated HUVECs (7.8 ± 0.8 nM); these effects were abrogated by anti‐TF IgG (15.9 ± 1.5 nM; Fig. 3D). In addition, pre‐incubation with compounds **1** and **2** resulted in a dose‐dependent inhibition of FX activation by FVIIa (Fig. 3D). The results of a chromogenic substrate assay demonstrate that compound **1** is a potent inhibitor of human thrombin and FXa with a *K*i∇″>−+ μM and ″>−° μM, respectively (Table 2). And, compound **2** is also a potent inhibitor of human thrombin and FXa with a *K*i∇″>×′ μM and ″>÷× μM, respectively (Table 2). Therefore, these results suggest that compounds **1** and **2** inhibit the production of thrombin and FXa.

**Table 2:** Enzyme kinetics of compounds 1 and 2

K_i_ a				
Enzyme	1	2	Argatroban	Rivaroxaban
α‐Thrombin	0.21 ± 0.043	0.39 ± 0.037	0.027 ± 0.0013	>250
Factor Xa	0.28 ± 0.031	0.43 ± 0.038	>250	0.004 ± 0.0028

a K_i_ is represented by the mean ± S.D. (*n* = 5), μM.

### Effects of the isolated compounds on the secretion of PAI‐1 and t‐PA

**Figure 4:**
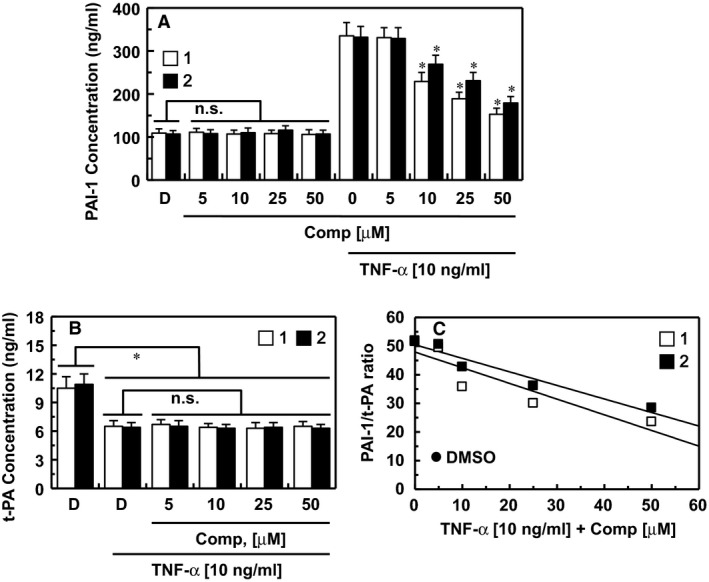
Effects of compounds 1 and 2 on the secretion of PAI‐1 and t‐PA. (**A**) HUVECs were cultured with compound **1** (white box) and compound **2** (black box) in the absence or presence of TNF‐α (10 ng/ml) for 18 hrs and the PAI‐1 concentrations in the culture media were determined as described in the ‘Materials and methods’ section. (**B**) HUVECs were cultured with each compound in the absence or presence of TNF‐α (10 ng/ml) for 18 hrs and the t‐PA concentrations in the culture media were determined as described in the ‘Materials and methods’ section. (**C**) The PAI‐1/t‐PA ratio in TNF‐α activated HUVECs from (**A**) and (**B**). *D* = 0.2% DMSO used as the vehicle control. *P < 0.05 *versus* TNF‐α or D alone; n.s., not significant.

TNF‐α is known to inhibit fibrinolysis in HUVECs by inducing the production of PAI‐I. The alteration of the balance between t‐PA and PAI‐1 is known to modulate coagulation and fibrinolysis 27. To determine the direct effects of each compound on TNF‐α‐stimulated secretion of PAI‐1, HUVECs were cultured in media with or without each compound and in the absence or presence of TNF‐α for 18 hrs. As shown in Figure 4A, treatment with compounds **1** and **2** resulted in a dose‐dependent inhibition of TNF‐α‐induced secretion of PAI‐1 from HUVECs, which was significant at 10‐50 μM.

TNF‐α does not have a significant effect on t‐PA production 28 and the balance between plasminogen activators and their inhibitors reflects the net plasminogen‐activating capacity 29; therefore, we investigated the effect of the combination of TNF‐α and compounds **1** and **2** on the secretion of t‐PA by HUVECs. Our results were consistent with those of a previous study reporting a modest decrease in the production of t‐PA by TNF‐α in HUVECs 30. This decrease was not significantly altered by treatment with compounds **1** and **2** (Fig. 4B). Collectively, these results indicate that TNF‐α increased the PAI‐1/t‐PA ratio, which was inhibited by compounds **1** and **2** (Fig. 4C).

### *In vivo* effects of the isolated compounds in an arterial thrombosis, a pulmonary thrombosis model and bleeding time

**Figure 5:**
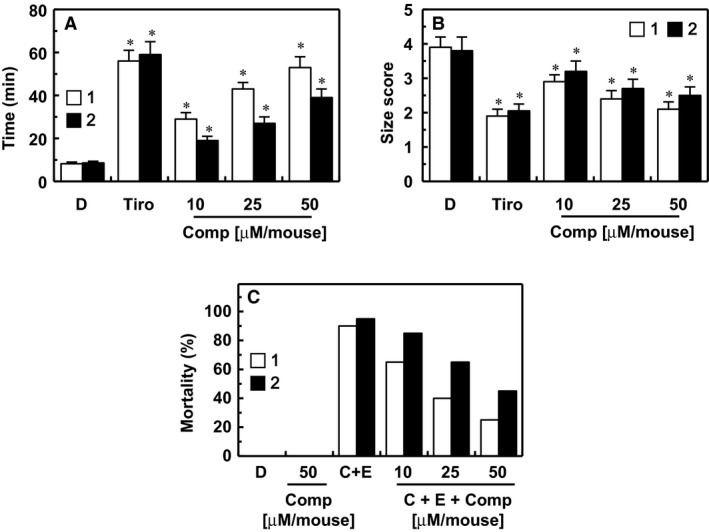
Effects of compounds 1 and 2 on arterial thrombosis and on acute thrombosis. (**A**) Time to large thrombus formation by compound **1** (white box) and compound **2** (black box). Tirofiban (Tiro) was used as a positive control. (**B**) The size score of the thrombus at 60 min. after FeCl_3_ treatment as described in ‘Materials and methods’. (**C**) After each compound was injected intravenously, a mixture of collagen (C, 500 μg/kg) plus epinephrine (E, 50 μg/kg) was injected into the tail vein of mice to induce acute thrombosis 6 hrs later. Then, mice (20 mice per group) were carefully examined for 15 min. to determine whether the mouse was paralysed, dead or recovered from the acute thrombotic challenge. *D* = 0.2% DMSO used as the vehicle control. *P < 0.05 *versus* DMSO.

The mouse model of ferric chloride (FeCl_3_)‐induced carotid artery thrombosis 31 has been commonly used to assess antiplatelet effects. The time to thrombus formation and the size of the resulting thrombi are summarized in Figure 5. Data showed that endothelial injury after FeCl_3_ treatment in control mice led to the growth of large thrombi at 8.2 ± 0.8 min. and the antiplatelet GP IIb/IIIa inhibitor, tirofiban, significantly slowed the growth of large thrombi at 54.1 ± 5.7 min. Compounds **1** and **2** significantly slowed the growth of thrombi (Fig. 5A). We also examined the effect of each compound on thrombus size at 60 min. after FeCl_3_‐induced endothelial injury (Fig. 5B). Results showed that compounds **1** and **2** reduced FeCl_3_‐induced thrombus formation. In addition, the results of the *in vivo* pulmonary thrombosis model are shown in Figure 5C. An intravenous injection of a mixture of collagen and epinephrine into mice induced massive pulmonary thrombosis, causing acute paralysis and sudden deaths (90–95% mortality). The mortalities in compound **1‐** and compound **2‐**treated groups decreased significantly compared to that in collagen‐ and epinephrine‐treated group (Fig. 5C).

To confirm above‐mentioned antithrombotic and antiplatelet functions, the *in vivo* tail bleeding times were determined. As shown in Table 1, the tail bleeding times were significantly prolonged by compounds **1** and **2** (i.v. injection) in comparison with those of the controls. The anticoagulation effect of each compound was observed *ex vivo* in mice as demonstrated by the dose‐dependent prolongation of the aPTT and PT (Table S4). However, the *ex vivo* coagulation time was not altered by other compounds **3**,** 4** and **5** (Table S4).

## Discussion

As a new biological resource in the development of the food and the pharmaceutical industries, the value of insects has been highly increased. Particularly, edible insects have attracted attention in the development of functional foods. The current study focused on the isolation of anticoagulant secondary metabolites from the larvae of *P. brevitarsis seulensis* and on the evaluation of isolated compounds by monitoring the clotting and bleeding time, platelet aggregation, and production of thrombin and FXa. Five indole alkaloids, 5‐hydroxyindolin‐2‐one (**1**), (1*R*,3*S*)‐1‐methyl‐1,2,3,4‐tetrahydro‐β‐carboline‐3‐carboxylic acid (**2**), (1*S*,3*S*)‐1‐methyl‐1,2,3,4‐tetrahydro‐β‐carboline‐3‐carboxylic acid (**3**), (3*S*)‐1,2,3,4‐tetrahydro‐β‐carboline‐3‐carboxylic acid (**4**) and L‐tryptophan (**5**) were isolated from the larvae. Compound **1** was synthetically reported 32, and compounds **2** and **3** were discovered from plants 20. However, this is the first report to show that they are chemical constituents of insects and that they exhibit antiplatelet activity. The indole alkaloid **1** was demonstrated to have a more potent antithrombotic activity than heparin. Tetrahydro‐β‐carbolines **2‐4** could be produced through a Pictet–Spengler condensation of tryptophan with aldehydes or α‐oxo acids 33. Although the antiplatelet activity of one of the tetrahydro‐β‐carbolines has been reported 34, there have been no reports on the anticoagulant effect of each diastereoisomers. In our evaluation, compound **2** (1*R*,3*S* diastereomer) displayed potent anticoagulant effect, whereas compound **3** (1*S*,3*S* diastereomer) did not.

The pre‐clinical evaluation of the antithrombotic potential of novel molecules requires the use of reliable and reproducible experimental animal preparations of thrombosis. The use of a highly sensitive intravital microscopic technique to study thrombus formation in real time has enabled us to establish a role for this molecule in the regulation of thrombus formation *in vivo*. One of the most widely used procedures employs topical application of ferric chloride (FeCl_3_) to an artery 35. The mouse model of FeCl_3_‐induced carotid artery thrombosis is an arterial thrombosis and a pulmonary thrombosis model and one of the most established and commonly used preparations to determine the efficacy of novel antithrombotic drugs *in vivo* 31. This is a simple and well‐established model known to be sensitive to both anticoagulant and antiplatelet drugs 35. In our experiment, a mouse model of acute arterial thrombosis was developed by applying FeCl_3_ to the outer tissues of arteries that had lost endothelial cell protection due to circulating platelets and components of the coagulation cascade, which was used to find both anticoagulant and antiplatelets drugs 36. A hallmark of the FeCl_3_ injury model is its sensitivity to thrombin inhibitors 37. In this study, the FeCl_3_ injury model produced an occlusive thrombosis quickly and stably (Fig. 5), which was suppressed by administering compounds **1** and **2**.

The PT, aPTT and platelet aggregation are the most established and commonly used methods to determine the efficacy of novel antithrombotic drugs 38. In our experiments, the PT and aPTT assays were performed using human plasma to evaluate the antithrombotic effects of compounds **1** and **2**, while platelet aggregation was evaluated to determine the antiplatelet activity of compounds **1** and **2**. LMWH and heparin could effectively inhibit aPPT and aPTT, and PT, respectively, and compounds **1** and **2** prolonged the PT and aPTT (Table 1). Furthermore, the antithrombotic effect of compound **1** was better than that of LMWH and heparin, indicating that compound **1** might be used as a novel strong antithrombotic drug. In addition, compounds **1** and **2** caused a significant inhibited platelet aggregation (Fig. 1).

Mode of action of compounds **1** and **2** for antithrombotic and antiplatelet effects was inhibiting (*i*) platelet aggregation, (*ii*) phosphorylation of MARCKS by PKC pathway, (*iii*) cytosolic Ca^2+^ mobilization, (*iv*) the activation and production of thrombin and FXa, (*v*) intrinsic and extrinsic coagulation times, and (*vi*) reducing PAI‐1/t‐PA ratio. The mechanism of coagulation involves activation, adhesion and aggregation of platelets 39, 40, 41. Coagulation begins almost instantly after an injury to the blood vessel has damaged the endothelium lining the vessel. Exposure of blood to the space under the endothelium initiates two processes: changes in platelets and the exposure of subendothelial tissue factor (TF) to factor VII (FVII), which ultimately leads to fibrin formation 39, 40, 41. Disruption of the endothelium exposes platelets to collagen in the vessel wall and FVIIa to TF. Subsequently, propagation of the thrombus involves recruitment of additional platelets and amplification of the coagulation cascade by the intrinsic pathway of blood coagulation, which includes the haemophilia factors FVIII and FIX 41. Importantly, platelets and endothelial cells play a critical role in the amplification of the coagulation cascade by providing a thrombogenic surface. Therefore, the activation and behaviours of platelets are the mechanistic targets of both pathways of coagulation and aggregation, suggesting that the inhibitory effects of compounds **1** and **2** on the activation and behaviours of platelets could be the most important functions of antithrombotic and antiplatelet effects of compounds **1** and **2**.

Platelets are activated by several agonists that bind to specific platelet membrane receptors 42. Serotonin (5‐hydroxytryptamine, 5‐HT) facilitates the development of platelets with increased pro‐coagulant activity and potentiates platelet activation, resulting in converging signal transduction pathways that would be responsible for blood coagulation 43, 44. This implies that modulation of serotonin‐mediated responses may offer a therapeutic target for the development of anticoagulant agent. Among the known 5‐HT receptors, 5‐HT_2A_ (formerly termed 5‐HT_2_) receptors on vascular smooth muscle and platelets play important roles in the cardiovascular system 45, 46, 47. Several studies suggest a connection between serotonergic mechanisms and cardiovascular events. Nishihira *et al*., reported that intravenous injection of sarpogrelate, an inhibitor of the 5‐HT_2A_ receptor (serotonin receptor), in a rabbit model significantly reduced the *ex vivo* platelet aggregation induced by ADP, thrombin and collagen alone as well as with 5‐HT and significantly prevented occlusive thrombus formation *in vivo* 48. Another study also reported that increased platelet 5‐HT could be effective in the control of bleeding in idiopathic thrombocytopenic purpura 49. Considering the structure of compounds **1** and **2** possessing indole core structure of serotonin, they may act as serotonin antagonists in platelet aggregation and thrombus formation.

As a commercial anticoagulant, heparin has been used for the prevention of venous thromboembolic diseases for more than 60 years 50. However, heparin has side effects such as the inability to inhibit fibrin‐bound thrombin activity, ineffectiveness in congenital or acquired antithrombin deficiencies, the development of thrombocytopenia, an increased risk of thromboembolic disease if the therapeutic response is not achieved and an increased risk of bleeding if the therapeutic range is exceeded 51. Furthermore, the amounts of available heparin are low in bovine lungs or pig intestines, from which heparin is primarily extracted 51. Therefore, the need for discovering alternative sources of anticoagulants has increased owing to the demand for a safer anticoagulant therapy. Based on the current findings, compounds **1** and **2** may provide new chemotypes for the development of anticoagulants provided their therapeutic effects are established.

In conclusions, this study demonstrated that compounds **1** and **2** inhibited the blood coagulation pathways by inhibiting FXa and thrombin production in HUVECs. They also inhibited the TNF‐α‐induced secretion of PAI‐1 and platelet aggregation *in vitro* and *ex vivo*. These results add to those from previous works on this topic and may be of interest to those designing pharmacological strategies for the treatment or prevention of coagulation‐related vascular diseases.

## Competing financial interests

The authors declare no competing financial interests.

## Supporting information


**Data S1** Supplementary Materials and Methods.
**Table S1** ^1^H and ^13^C NMR Spectroscopic Data for Compound 1 (^1^H: 600 MHz; ^13^C: 150 MHz; in Acetone‐*d*_*6*_).
**Table S2** ^1^H and ^13^C NMR Spectroscopic Data for Compounds 2 and 3 (^1^H: 600 MHz; ^13^C: 150 MHz; in D_2_O and DMSO‐*d*_*6*_).
**Table S3** Anticoagulant activity of other compounds.
**Table S4** *Ex vivo* coagulation time of compounds.
**Figure S1** Compounds isolated from *Protaetia brevitarsis seulensis* (Kolbe).
**Figure S2** Key HMBC (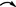) correlations of compound 1 and 2.
**Figure S3** HRESIMS spectrum of compound **1**.
**Figure S4** ^1^H NMR of compound **1** (600 MHz, acetone‐*d*_*6*_).
**Figure S5** ^13^C NMR spectrum of compound **1** (150 MHz, acetone‐*d*_*6*_).
**Figure S6** HSQC data of compound 1.
**Figure S7** HMBC data of compound 1.
**Figure S8** ^1^H NMR spectrum of compound **2** (600 MHz, D_2_O).
**Figure S9** ^13^C NMR spectrum of compound **2** (150 MHz, D_2_O).
**Figure S10** HSQC data of compound 2.
**Figure S11** HMBC data of compound 2.
**Figure S12** ^1^H NMR spectrum of compound **3** (600 MHz, DMSO‐*d*_*6*_).
**Figure S13** ^13^C NMR spectrum of compound **3** (150 MHz, DMSO‐*d*_*6*_).
**Figure S14** ^1^H NMR spectrum of compound **4** (600 MHz, DMSO‐*d*_*6*_).
**Figure S15** ^1^H NMR spectrum of compound **5** (600 MHz, DMSO‐*d*_*6*_).

## References

[1] Fares A . Winter cardiovascular diseases phenomenon. N Am J Med Sci. 2013; 5: 266–79. 23724401 10.4103/1947-2714.110430 PMC3662093

[2] Davi G , Patrono C . Platelet activation and atherothrombosis. N Engl J Med. 2007; 357: 2482–94. 18077812 10.1056/NEJMra071014

[3] Fuster V , Bhatt DL , Califf RM , *et al* Guided antithrombotic therapy: current status and future research direction: report on a National Heart, Lung and Blood Institute working group. Circulation. 2012; 126: 1645–62. 23008471 10.1161/CIRCULATIONAHA.112.105908 PMC4086864

[4] Bell S , Nand J , Spriggs D . New antithrombotic drugs for atrial fibrillation: caution is needed. Lancet. 2012; 9813: e24; author reply e‐5. 10.1016/S0140-6736(12)60146-9 22284660

[5] Coccheri S . Antiplatelet drugs–do we need new options? With a reappraisal of direct thromboxane inhibitors. Drugs. 2010; 70: 887–908. 20426498 10.2165/11536000-000000000-00000

[6] Koenig‐Oberhuber V , Filipovic M . New antiplatelet drugs and new oral anticoagulants. Br J Anaesth. 2016; 117: ii74–84. 27566810 10.1093/bja/aew214

[7] Lee W , Lee J , Kulkarni R , *et al* Antithrombotic and antiplatelet activities of small‐molecule alkaloids from *Scolopendra subspinipes mutilans* . Sci Rep. 2016; 6: 21956. 26905699 10.1038/srep21956 PMC4764974

[8] Shi YN , Tu ZC , Wang XL , *et al* Bioactive compounds from the insect *Aspongopus chinensis* . Bioorg Med Chem Lett. 2014; 24: 5164–9. 25442305 10.1016/j.bmcl.2014.09.083

[9] Ebaid H , Al‐Tamimi J , Hassan I , *et al* Antioxidant bioactivity of samsum Ant (*Pachycondyla sennaarensis*) venom protects against CCL4‐induced nephrotoxicity in mice. Oxid Med Cell Longev. 2014; 2014: 8. 10.1155/2014/763061 PMC3997132 24803985

[10] Lee JE , Lee AJ , Jo DE , *et al* Cytotoxic effects of *Tenebrio molitor* larval extracts against hepatocellular carcinoma. J Korean Soc Food Sci Nutr. 2015; 44: 200–7.

[11] Kim HG , Park K‐H , Lee S , *et al* Comparison of clay and charcoal as feed additives for *Protaetia brevitarsis* (Coleoptera: Scarabaeidae). Int J Indust Entomol. 2015; 31: 25–9.

[12] Kwon O . Effect of different diets on larval growth of *Protaetia brevitarsis seulensis* (Kolbe) (Coleoptera: Cetoniidae). Entomol Res. 2009; 39: 152–4.

[13] Yoon HS , Lee CS , Lee SY , *et al* Purification and cDNA cloning of inducible antibacterial peptides From *Protaetia brevitarsis* (Coleoptera). Arch Insect Biochem Physiol. 2003; 52: 92–103. 12529864 10.1002/arch.10072

[14] Yeo H , Youn K , Kim M , *et al* Fatty acid composition and volatile constituents of *Protaetia brevitarsis* larvae. Prev Nutr Food Sci. 2013; 18: 150–6. 24471125 10.3746/pnf.2013.18.2.150 PMC3892504

[15] Yoo YC , Shin BH , Hong JH , *et al* Isolation of fatty acids with anticancer activity from *Protaetia brevitarsis* larva. Arch Pharm Res. 2007; 30: 361–5. 17424944 10.1007/BF02977619

[16] Lee W , Seo J , Kwak S , *et al* A double‐chambered protein nanocage loaded with thrombin receptor agonist Peptide (TRAP) and gamma‐carboxyglutamic acid of protein C (PC‐Gla) for sepsis treatment. Adv Mater. 2015; 27: 6637–43. 26414883 10.1002/adma.201503093

[17] Kim SY , Kim S , Kim JM , *et al* PKC inhibitors RO 31‐8220 and Go 6983 enhance epinephrine‐induced platelet aggregation in catecholamine hypo‐responsive platelets by enhancing Akt phosphorylation. BMB Rep. 2011; 44: 140–5. 21345315 10.5483/BMBRep.2011.44.2.140

[18] Lee W , Ku SK , Bae JS . Antiplatelet, anticoagulant, and profibrinolytic activities of baicalin. Arch Pharm Res. 2015; 38: 893–903. 24849036 10.1007/s12272-014-0410-9

[19] Kim TH , Ku SK , Bae JS . Antithrombotic and profibrinolytic activities of eckol and dieckol. J Cell Biochem. 2012; 113: 2877–83. 22511271 10.1002/jcb.24163

[20] Wang X , Liu R , Yang Y , *et al* Isolation, purification and identification of antioxidants in an aqueous aged garlic extract. Food Chem. 2015; 187: 37–43. 25976995 10.1016/j.foodchem.2015.03.109

[21] Diehl KH , Hull R , Morton D , *et al* A good practice guide to the administration of substances and removal of blood, including routes and volumes. J Appl Toxicol. 2001; 21: 15–23. 11180276 10.1002/jat.727

[22] Wei AH , Schoenwaelder SM , Andrews RK , *et al* New insights into the haemostatic function of platelets. Br J Haematol. 2009; 147: 415–30. 19656155 10.1111/j.1365-2141.2009.07819.x

[23] Elzagallaai A , Rose SD , Trifaro JM . Platelet secretion induced by phorbol esters stimulation is mediated through phosphorylation of MARCKS: a MARCKS‐derived peptide blocks MARCKS phosphorylation and serotonin release without affecting pleckstrin phosphorylation. Blood. 2000; 95: 894–902. 10648401

[24] Sugo T , Nakamikawa C , Tanabe S , *et al* Activation of prothrombin by factor Xa bound to the membrane surface of human umbilical vein endothelial cells: its catalytic efficiency is similar to that of prothrombinase complex on platelets. J Biochem. 1995; 117: 244–50. 7608107 10.1093/jb/117.2.244

[25] Rao LV , Rapaport SI , Lorenzi M . Enhancement by human umbilical vein endothelial cells of factor Xa‐catalyzed activation of factor VII. Blood. 1988; 71: 791–6. 3422831

[26] Ghosh S , Ezban M , Persson E , *et al* Activity and regulation of factor VIIa analogs with increased potency at the endothelial cell surface. J Thromb Haemost. 2007; 5: 336–46. 17092304 10.1111/j.1538-7836.2007.02308.x

[27] Philip‐Joet F , Alessi MC , Philip‐Joet C , *et al* Fibrinolytic and inflammatory processes in pleural effusions. Eur Respir J. 1995; 8: 1352–6. 7489803 10.1183/09031936.95.08081352

[28] Hamaguchi E , Takamura T , Shimizu A , *et al* Tumor necrosis factor‐alpha and troglitazone regulate plasminogen activator inhibitor type 1 production through extracellular signal‐regulated kinase‐ and nuclear factor‐kappaB‐dependent pathways in cultured human umbilical vein endothelial cells. J Pharmacol Exp Ther. 2003; 307: 987–94. 14534369 10.1124/jpet.103.054346

[29] Quinn C , Hill J , Hassouna H . A guide for diagnosis of patients with arterial and venous thrombosis. Clin Lab Sci. 2000; 13: 229–38. 11586510

[30] Lopez S , Peiretti F , Bonardo B , *et al* Effect of atorvastatin and fluvastatin on the expression of plasminogen activator inhibitor type‐1 in cultured human endothelial cells. Atherosclerosis. 2000; 152: 359–66. 10998463 10.1016/s0021-9150(00)00454-8

[31] Izuhara Y , Takahashi S , Nangaku M , *et al* Inhibition of plasminogen activator inhibitor‐1: its mechanism and effectiveness on coagulation and fibrosis. Arterioscler Thromb Vasc Biol. 2008; 28: 672–7. 18239154 10.1161/ATVBAHA.107.157479

[32] Sinananwanich W , Segawa Y **,** Higashihara T , *et al* Base‐Catalyzed Synthesis of a 100% Hyperbranched Polymer on the Basis of an Indolin‐2‐one Unit. Macromolecules. 2009; 42: 8718–24.

[33] Rashid N , Alam S , Hasan M , *et al* Cis‐diastereoselectivity in pictet–spengler reactions of L‐tryptophan and electronic circular dichroism studies. Chirality. 2012; 24: 789–95. 22760664 10.1002/chir.22070

[34] Tsuchiya H , Sato M , Watanabe I . Antiplatelet activity of soy sauce as functional seasoning. J Agric Food Chem. 1999; 47: 4167–74. 10552785 10.1021/jf990147d

[35] Sachs UJ , Nieswandt B . *In vivo* thrombus formation in murine models. Circ Res. 2007; 100: 979–91. 17431199 10.1161/01.RES.0000261936.85776.5f

[36] Li W , Febbraio M , Reddy SP , *et al* CD36 participates in a signaling pathway that regulates ROS formation in murine VSMCs. J Clin Invest. 2010; 120: 3996–4006. 20978343 10.1172/JCI42823 PMC2964976

[37] Lyle EM , Lewis SD , Lehman ED , *et al* Assessment of thrombin inhibitor efficacy in a novel rabbit model of simultaneous arterial and venous thrombosis. Thromb Haemost. 1998; 79: 656–62. 9531058

[38] Jauch EC , Saver JL , Adams HP Jr , *et al* Guidelines for the early management of patients with acute ischemic stroke: a guideline for healthcare professionals from the American Heart Association/American Stroke Association. Stroke. 2013; 44: 870–947. 23370205 10.1161/STR.0b013e318284056a

[39] Furie B , Furie BC . Thrombus formation *in vivo* . J Clin Invest. 2005; 115: 3355–62. 16322780 10.1172/JCI26987 PMC1297262

[40] Sim D , Flaumenhaft R , Furie B . Interactions of platelets, blood‐borne tissue factor, and fibrin during arteriolar thrombus formation *in vivo* . Microcirculation. 2005; 12: 301–11. 15814438 10.1080/10739680590925682

[41] Dahlback B . Blood coagulation. Lancet. 2000; 355: 1627–32. 10821379 10.1016/S0140-6736(00)02225-X

[42] Rivera J , Lozano ML , Navarro‐Nunez L , *et al* Platelet receptors and signaling in the dynamics of thrombus formation. Haematologica. 2009; 94: 700–11. 19286885 10.3324/haematol.2008.003178 PMC2675683

[43] Vanags DM , Rodgers SE , Duncan EM , *et al* Potentiation of ADP‐induced aggregation in human platelet‐rich plasma by 5‐hydroxytryptamine and adrenaline. Br J Pharmacol. 1992; 106: 917–23. 1393289 10.1111/j.1476-5381.1992.tb14435.x PMC1907675

[44] Bampalis VG , Dwivedi S , Shai E , *et al* Effect of 5‐HT2A receptor antagonists on human platelet activation in blood exposed to physiologic stimuli and atherosclerotic plaque. J Thromb Haemost. 2011; 9: 2112–5. 21848639 10.1111/j.1538-7836.2011.04476.x

[45] de Clerck F , David JL , Janssen PA . Inhibition of 5‐hydroxytryptamine‐induced and ‐amplified human platelet aggregation by ketanserin (R 41 468), a selective 5‐HT2‐receptor antagonist. Agents Actions. 1982; 12: 388–97. 6215842 10.1007/BF01965409

[46] Nilsson T , Longmore J , Shaw D , *et al* Characterisation of 5‐HT receptors in human coronary arteries by molecular and pharmacological techniques. Eur J Pharmacol. 1999; 372: 49–56. 10374714 10.1016/s0014-2999(99)00114-4

[47] Wiernsperger N . Serotonin 5‐HT2 receptors and brain circulation. J Cardiovasc Pharmacol. 1990; 16: S20–4. 1369712

[48] Nishihira K , Yamashita A , Tanaka N , *et al* Inhibition of 5‐hydroxytryptamine(2A) receptor prevents occlusive thrombus formation on neointima of the rabbit femoral artery. J Thromb Haemost. 2006; 4: 247–55. 16409475 10.1111/j.1538-7836.2005.01702.x

[49] Lechin F , van der Dijs B , Orozco B , *et al* Neuropharmacological treatment of refractory idiopathic thrombocytopenic purpura: roles of circulating catecholamines and serotonin. Thromb Haemost. 2004; 91: 1254–6. 15175817

[50] Wells PS , Forgie MA , Rodger MA . Treatment of venous thromboembolism. JAMA. 2014; 311: 717–28. 24549552 10.1001/jama.2014.65

[51] Pereira MS , Melo FR , Mourao PA . Is there a correlation between structure and anticoagulant action of sulfated galactans and sulfated fucans? Glycobiology. 2002; 12: 573–80. 12244069 10.1093/glycob/cwf077

