# Supplementary material for: Inhibition of platelet aggregation and thrombosis by indole alkaloids isolated from the edible insect Protaetia brevitarsis seulensis (Kolbe)
Source: J Cell Mol Med. 2016 Dec 20;21(6):1217–27. doi: 10.1111/jcmm.13055 (PMC5431138; doi:10.1111/jcmm.13055)
Supplement: Supplementary file 1 — Data S1 Supplementary Materials and Methods. Table S1 1H and 13C NMR Spectroscopic Data for Compound 1 (1H: 600 MHz; 13C: 150 MHz; in Acetone‐d 6). Table S2 1H and 13C NMR Spectroscopic Data for Compounds 2 and 3 (1H: 600 MHz; 13C: 150 MHz; in D2O and DMSO‐d 6). Table S3 Anticoagulant activity of other compounds. Table S4 Ex vivo coagulation time of compounds. Figure S1 Compounds isolated from Protaetia brevitarsis seulensis (Kolbe). Figure S2 Key HMBC () correlations of compound 1 and 2. Figure S3 HRESIMS spectrum of compound 1. Figure S4 1H NMR of compound 1 (600 MHz, acetone‐d 6). Figure S5 13C NMR spectrum of compound 1 (150 MHz, acetone‐d 6). Figure S6 HSQC data of compound 1. Figure S7 HMBC data of compound 1. Figure S8 1H NMR spectrum of compound 2 (600 MHz, D2O). Figure S9 13C NMR spectrum of compound 2 (150 MHz, D2O). Figure S10 HSQC data of compound 2. Figure S11 HMBC data of compound 2. Figure S12 1H NMR spectrum of compound 3 (600 MHz, DMSO‐d 6). Figure S13 13C NMR spectrum of compound 3 (150 MHz, DMSO‐d 6). Figure S14 1H NMR spectrum of compound 4 (600 MHz, DMSO‐d 6). Figure S15 1H NMR spectrum of compound 5 (600 MHz, DMSO‐d 6). [file JCMM-21-1217-s001.doc]

# Inhibition of platelet aggregation and thrombosis by indole alkaloids from the edible insect *Protaetia brevitarsis seulensis* (Kolbe)

# JungIn Lee^1†^, Wonhwa Lee ^2†^, Mi-Ae Kim^2,3†^, Jae Sam Hwang^3^, MinKyun Na^1^*, and Jong-Sup Bae^2^*

^1^College of Pharmacy, Chungnam National University, Daejeon, 34134, Republic of Korea; ^2^College of Pharmacy, CMRI, Research Institute of Pharmaceutical Sciences, BK21 Plus KNU Multi-Omics based Creative Drug Research Team, Kyungpook National University, Daegu 41566 Republic of Korea; ^3^Department of Agricultural Biology, The National Academy of Agricultural Science, RDA, Wanju-gun, 55365, Republic of Korea

Running title: Anticoagulant effects of indole alkaloids

# ^†^These authors contributed equally to this work

# * Corresponding Authors:

# MinKyun Na, Ph.D.

College of Pharmacy, Chungnam National University

99 Daehak-ro, Yuseong, Daejeon 34134, Republic of Korea

# Phone, 82-42-821-5925; Fax, 82-42-823-6566

# Email: mkna@cnu.ac.kr

# Jong-Sup Bae, Ph.D.

College of Pharmacy, Kyungpook National University

# 80 Daehak-ro, Buk-gu, Daegu 41566, Republic of Korea

# Phone, 82-53-950-8570; Fax, 82-53-950-8557

# Email: baejs@knu.ac.kr

**Supplementary Materials and Methods**

**Reagents**

Freeze-dried individuals of *Protaetia brevitarsis seulensis* (Kolbe) (5.17 kg) were obtained from the National Academy of Agricultural Science, RDA, Korea, and identified by one of the authors (M-A. Kim). A voucher specimen (CNU-INS 1502) has been deposited at the Pharmacognosy Laboratory of the College of Pharmacy, Chungnam National University, Daejeon, Korea. TNF-α was purchased from Abnova (Taipei, Taiwan). The anti-tissue factor (TF) antibody was purchased from Santa Cruz Biotechnology, Inc. (Santa Cruz, CA, USA). The thromboxane A2 (TXA2) analogue U46619 was purchased from Calbiochem-Novabiochem Corp. (San Diego, CA, USA). Factor V, VII, VIIa, X, and Xa, and antithrombin III (AT III), prothrombin, and thrombin were obtained from Haematologic Technologies (Essex Junction, VT, USA). The aPTT assay reagent and PT reagents were purchased from Fisher Diagnostics (Middletown, VA, USA); the chromogenic substrates (S-2222 and S-2238) were purchased from Chromogenix AB (Mölndal, Sweden). Rivaroxaban and argatroban (a direct FXa and FIIa inhibitor, respectively) were purchased from Santa Cruz Biotechnology, Inc. (Dallas, TX, USA). The plasminogen activator inhibitor-1 (PAI-1) and tissue plasminogen activator (t-PA) enzyme-linked immunosorbent assay (ELISA) kits were purchased from American Diagnostica, Inc. (Stamford, CT, USA). Collagen was purchased from Sigma (St. Louis, MO, USA). All other reagents were of the highest commercially available grade.

**Isolation of human plasma and platelets**

Human blood samples were collected in the morning from 10 healthy, fasted volunteers (age: 24-28 years, four males and six females) without cardiovascular disorders, allergy and lipid or carbohydrate metabolism disorders, and who did not received drug treatment. All subjects gave written informed consent before participation in the study. The subjects did not use addictive substances or antioxidant food supplementation, and their diet was balanced (meat and vegetables). Blood was collected in sodium citrate (0.32% final concentration, 10.9 mM) and immediately centrifuged (1,300 g × 15 min) in order to obtain plasma and pooled plasma was used for further study. Human platelets were prepared as described previously [1-2]. Briefly, platelet rich plasma (PRP) was prepared by centrifugation at room temperature for 15 min at 150 g. PRP was adjusted to a concentration of 1 x 10^9^ platelets/mL with use of a hemocytometer for cell counts. PRP was washed once with HEPES buffer (5 mM HEPES, 136 mM NaCl, 2.7 mM KCl, 0.42 mM NaH_2_PO_4_, 2 mM MgCl_2_, 5.6 mM glucose, 0.1% BSA (w/v), pH to 7.45) in the presence of 1mM CaCl_2_. The platelets were left at room temperature for 30 min. Ten-milliliter blood samples were used for each clotting time point measurement. The study protocol (KNUH 2012-01-010) was approved by the Institutional Review Board of Kyungpook National University Hospitals (Daegu, Republic of Korea).

**Animals and husbandry**

Male C57BL/6 mice (6-7 weeks old, weighing 27 g) were purchased from Orient Bio Co. (Sungnam, Republic of Korea) and were used after a 12-day acclimatization period. The mice were housed at five per polycarbonate cage under a controlled temperature (20-25°C) and humidity (40-45% relative humidity) and a 12:12 h light:dark cycle. They received a normal rodent-pellet diet and water *ad libitum* during acclimatization and were treated in accordance with the Guidelines for the Care and Use of Laboratory Animals issued by Kyungpook National University, Republic of Korea (IRB No. KNU 2016-54).

**Determination of *in vitro* enzyme inhibition**

The inhibitor constants (K_i_) were determined for inhibition by each compound, argatroban, and rivaroxaban. Chromogenic substrate assays were performed using a Labsystems IEMS (Cergy Pontoise, France) microtiter plate reader. K_i_ values were calculated according to the method of Dixon [3]. In each assay, the compound was tested at a minimum of seven concentrations in duplicate to obtain an inhibition curve. Assays were performed according to the following general procedure. In a 96-well microtiter plate, 25 μL of compound, inhibitor solution or buffer was added to 50 μL of substrate. A volume of 25 μL of enzyme solution was added just before the plate was placed in the microtiter plate reader for 1 h at 37°C. The hydrolysis of the substrate yields *p*-nitroaniline, which was continuously monitored spectrophotometrically at 405 nm. Maximal initial reaction rates were calculated and expressed as millioptical density per minute. Curve fitting (Dixon plot of 1/V_max_ versus inhibitor concentration) was performed by linear regression analysis to calculate the K_i_ value.

**PAI-1 and t-PA ELISA**

The concentrations of PAI-1 and t-PA in HUVEC cultured supernatants were determined using ELISA kits (American Diagnostica, Inc., Stamford, CT, USA).

**Arterial thrombosis animal model**

The FeCl_3_-induced thrombosis mouse model was established as previously described [4]. Male C57BL/6 mice were fasted overnight and were administered each indicated compound in DMSO by intravenous injection. Then, mice were anesthetized using 3% isoflurane (Forane®, Choongwae Pharma. Corp., Seoul, Korea) and injected intravenously with 0.1 mL of 0.1% rhodamine 6G (Sigma). A testicular artery (200 μm in diameter) was carefully exposed and a cotton thread (0.2 mm in diameter) saturated with 0.25 mol/L FeCl_3_ was applied to the adventitial surface. After 5 min, the cotton thread was removed, and the wound was flushed with saline solution. Thrombus formation was monitored at 35°C by 3-dimensional imaging as previously described [5]. The size and time of thrombus formation were monitored, and the findings were categorized as follows: score 0 indicates no thrombus; 1 indicates small thrombus (50 μm × 75 μm); 2 indicates medium-sized thrombus (100 μm × 150 μm); and 3 indicates large thrombus (200 μm × 300 μm). The time from FeCl_3_-mediated endothelial injury to occlusion of the testicular artery by a large thrombus was measured.

**Acute thrombosis induced by a combination of collagen and epinephrine in mice**

Male C57BL/6 mice were fasted overnight and divided into groups of 10 animals. Each compound suspended in DMSO was administered to mice intravenously. A mixture of collagen (500 μg/kg) plus epinephrine (50 μg/kg) was injected into the tail vein of mice to induce acute thrombosis 1 h later. Each mouse was carefully examined for 15 min to determine whether the mouse was paralyzed, dead, or recovered from the acute thrombotic challenge. For statistical analysis, five separated experiments were performed.

**Western blotting**

Total cell extracts were prepared by lysis of the HUVECs and the protein concentration was determined using Bradford assay. Equal amounts of protein were separated by SDS-PAGE (10%) and electroblotted overnight onto an Immobilon membrane (Millipore, Billerica, MA, USA). The membranes were blocked for 1 h with 5% low-fat milk powder in TBS (50 mM Tris-HCl, pH 7.5, 150 mM NaCl) containing 0.05% Tween® 20. They were subsequently incubated with phospho-MARCKS (Santa Cruz, CA, USA) for 1.5 h at room temperature, followed by incubation with horseradish-peroxidase-conjugated secondary antibody and ECL-detection according to the manufacturer's instructions and as previously described [6]. β-actin (1:1000, Santa Cruz, CA, USA) was used as a loading control.

**Measurement of the intracellular Ca^2+^ mobilization**

The intracellular Ca^2+^ mobilization ([Ca^2+^]_i_) of platelets was measured as described previously [7]. Briefly, platelets were incubated with fura-2/AM (3 μM) at 37°C for 30 min. After washing twice, the fura-2-loaded platelets were suspended in Ca^2+^-free Tyrode's solution at a final concentration of 5 × 10^7^ platelets/mL. Calcium (1 mM) was added to the fura-2-loaded platelets 1 min before stimulation with platelet activators. The fluorescence (Ex 339 nm, Em 500 nm) was measured with a fluorescence spectrophotometer (TECAN, Männedorf, Switzerland). The [Ca^2+^]_i_ was calculated using the equation described by Grynkiewicz et al. [8].

**Production of factor Xa on the surface of HUVECs**

The TNF-α-stimulated (10 ng/mL for 6 h in serum-free medium) confluent monolayer of HUVECs (preincubated with the indicated concentrations of each compound for 10 min) in a 96-well culture plate was incubated with FVIIa (10 nM) in buffer B (buffer A [10 mM HEPES, pH 7.45, 150 mM NaCl, 4 mM KCl, and 11 mM glucose] supplemented with 5 mg/mL bovine serum albumin [BSA] and 5 mM CaCl_2_) for 5 min at 37°C in the presence or absence of anti-TF IgG (25 μg/mL). FX (175 nM) was subsequently added to the cells in a final reaction mixture volume of 100 μL, and the cells were incubated for 15 min. The reaction was stopped by the addition of buffer A containing 10 mM EDTA and the amounts of FXa generated were measured using a chromogenic substrate. The changes in absorbance at 405 nm over 2 min were monitored using a microplate reader (Tecan Austria GmbH, Grödig, Austria). The initial color development rates were converted into FXa concentrations using a standard curve prepared with known dilutions of purified human FXa.

**Production of thrombin on the surfaces of HUVECs**

Thrombin production by HUVECs was quantified as previously described [9-10]. Briefly, HUVECs were pre-incubated in 300 μL containing each compound in 50 mM Tris-HCl buffer, 100 pM FVa, and 1 nM FXa for 10 min, followed by the addition of prothrombin to a final concentration of 1 μM. After 10 min, duplicate samples (10 μL each) were transferred into a 96-well plate containing 40 μL of 0.5 M EDTA in TBS per well to terminate the prothrombin activation. Activated prothrombin was determined by measuring the rate of hydrolysis of S-2238 (a thrombin substrate) at 405 nm. Standard curves were prepared with known amounts of purified thrombin.

**Statistical Analysis**

The results were expressed as the means ± standard error of the mean (SEM) of at least three independent experiments performed in duplicate. *P* < 0.05 was considered statistically significant and was determined using the SPSS software (version 14.0, SPSS Science, Chicago, IL, USA). Statistical relevance was determined by one-way analysis of variance (ANOVA) and Tukey’s post-test.

**Supplementary Table 1. ^1^H and ^13^C NMR Spectroscopic Data for Compound 1 (^1^H: 600 MHz; ^13^C: 150 MHz; in Acetone-*d_6_*)**

|  |  | **1** | |
| --- | --- | --- | --- |
| Position |  | ^1^H (*J* in Hz) | ^13^C, type |
| 1 |  | - | - |
| 2 |  | - | 176.5, C |
| 3 |  | 3.37 (s) | 36.5, CH_2_ |
| 3a |  | - | 127.9, C |
| 4 |  | 6.78 (d, 2.1) | 113.4, CH |
| 5 |  | - | 153.5, C |
| 6 |  | 6.65 (d, 8.3, 2.1) | 114.4, CH |
| 7 |  | 6.70 (d, 8.3) | 110.3, CH |
| 7a |  | - | 136.9, C |
| -NH |  | 9.08 (brs) | - |
| -OH |  | 7.95 (brs) | - |

**Supplementary Table 2. ^1^H and ^13^C NMR Spectroscopic Data for Compounds 2 and 3**

**(^1^H: 600 MHz; ^13^C: 150 MHz; in D_2_O and DMSO-*d_6_*)**

|  |  | **2** | | **3** | |
| --- | --- | --- | --- | --- | --- |
| Position |  | ^1^H (*J* in Hz) | ^13^C, type | ^1^H (*J* in Hz) | ^13^C, type |
| 1 |  | 4.99 (dd, 13.7, 6.8) | 47.6, CH | 4.52 (d, 6.2) | 49.0, CH |
| 2 |  | - | - | 11.08 (s) | - |
| 3 |  | 4.26 (dd, 9.2, 5.5) | 52.9, CH | 3.63 (dd, 11.9, 4.4) | 57.6, CH |
| 4 |  | 3.40 (dd, 16.4, 5.5)  3.12 (ddd, 16.4, 9.2, 0.9), | 21.9, CH_2_ | 3.17 (dd, 16.0, 4.4)  2.78 (m) | 23.2, CH_2_ |
| 4a |  | - | 104.6 | - | 106.7, C |
| 4b |  | - | 125.3 | - | 126.1, C |
| 5 |  | 7.62 (d, 7.7) | 118.1 | 7.45 (d, 8.0) | 118.0, CH |
| 6 |  | 7.27 (t, 7.7) | 119.5 | 7.00 (m) | 118.9, CH |
| 7 |  | 7.18 (t, 7.7) | 122.4 | 7.09 (m) | 121.4, CH |
| 8 |  | 7.47 (d, 7.7) | 111.4 | 7.34 (d, 8.0) | 111.2, CH |
| 8a |  | - | 136.2 | - | 136.4, C |
| 9 |  | - | - | - | - |
| 9a |  | - | 130.4 | - | 132.2, C |
| 10 |  | 1.69 (d, 6.9) | 17.4 | 1.61 (d, 6.8) | 16.9, CH_3_ |
| -COOH |  | - | 173.6 | - | 169.5 |

**Supplementary Table 3. Anticoagulant activity of other compounds.**

| *In vitro* coagulant assay | | | | |
| --- | --- | --- | --- | --- |
| Sample | Dose | aPTT (s) | PT (s) | PT (INR) |
| Control | saline | 23.7 ± 0.4 | 11.7 ± 0.4 | 1.00 |
| **3** | 25 μM | 23.8 ± 0.2 | 11.8 ± 0.2 | 1.02 |
|  | 50 μM | 23.2 ± 0.4 | 11.9 ± 0.2 | 1.04 |
| **4** | 25 μM | 23.1 ± 0.3 | 11.7 ± 0.3 | 1.00 |
|  | 50 μM | 23.4 ± 0.4 | 12.2 ± 0.3 | 1.11 |
| **5** | 25 μM | 23.8 ± 0.3 | 11.1 ± 0.3 | 0.88 |
|  | 50 μM | 23.3 ± 0.5 | 11.5 ± 0.2 | 0.96 |
| LMWH | 10 IU/mL | 31.5 ± 0.6^*^ | 12.2 ± 0.4 | 1.11 |
| Heparin | 0.5 mg/mL | 57.8 ± 0.4^*^ | 26.7 ± 0.6^*^ | 7.24^*^ |

^a^Each value represents the means±SEM (n=5).

* p < 0.05 as compared to control.

**Supplementary Table 4. *Ex vivo* coagulation time of compounds.**

| Sample | Dose | aPTT (s) | PT (s) | PT (INR) |
| --- | --- | --- | --- | --- |
| Control | saline | 31.4 ± 0.4 | 12.2 ± 0.3 | 1.00 |
| Comp 1 | 7.5 μg/mouse | 51.7 ± 0.6^*^ | 25.4 ± 0.5^*^ | 5.81^*^ |
|  | 14.9 μg/mouse | 63.4 ± 1.2^*^ | 51.4 ± 1.4^*^ | 31.55^*^ |
| Comp 2 | 11.5 μg/mouse | 44.6 ± 0.5^*^ | 14.3 ± 0.6^*^ | 1.46^*^ |
|  | 23.0 μg/mouse | 51.7 ± 0.7^*^ | 24.2 ± 0.4^*^ | 5.17^*^ |
| **3** | 11.5 μg/mouse | 31.3 ± 0.6 | 12.4 ± 0.4 | 1.04 |
|  | 23.0 μg/mouse | 31.5 ± 1.0 | 12.3 ± 0.6 | 1.02 |
| **4** | 10.8 μg/mouse | 31.8 ± 0.7 | 12.5 ± 0.5 | 1.06 |
|  | 21.6 μg/mouse | 31.3 ± 0.5 | 12.6 ± 0.4 | 1.08 |
| **5** | 10.2 μg/mouse | 31.5 ± 0.4 | 12.4 ± 0.6 | 1.04 |
|  | 20.4 μg/mouse | 31.1 ± 0.5 | 12.0 ± 0.7 | 0.96 |

^a^ Each value represents the means±SEM (n=5).

* p < 0.05 as compared to control.

**Figure S1. Compounds isolated from *Protaetia brevitarsis seulensis* (Kolbe)**

**Figure S2. Key HMBC ( ) correlations of compounds 1 and 2.**

**Figure S3.** HRESIMS spectrum of compound **1**

**Elemental Composition Report**

Single Mass Analysis

Tolerance = 5.0 PPM / DBE: min = -1.5, max = 50.0

Element prediction: Off

Number of isotope peaks used for i-FIT = 3

Monoisotopic Mass, Even Electron Ions

21 formula(e) evaluated with 1 results within limits (all results (up to 1000) for each mass)

Elements Used:

C: 1-10 H: 1-10 N: 1-5 O: 1-5

Minimum: -1.5

Maximum: 5.0 5.0 50.0

Mass Calc. Mass mDa PPM DBE i-FIT Norm Conf(%) Formula

150.0554 150.0555 -0.1 -0.7 5.5 685.9 n/a n/a C8 H8 N O2

**Figure S4.** ^1^H NMR of compound **1** (600 MHz, acetone-*d_6_*)

**Figure S5**. ^13^C NMR spectrum of compound **1** (150 MHz, acetone-*d_6_*)

**Figure S6.** HSQC data of compound **1**


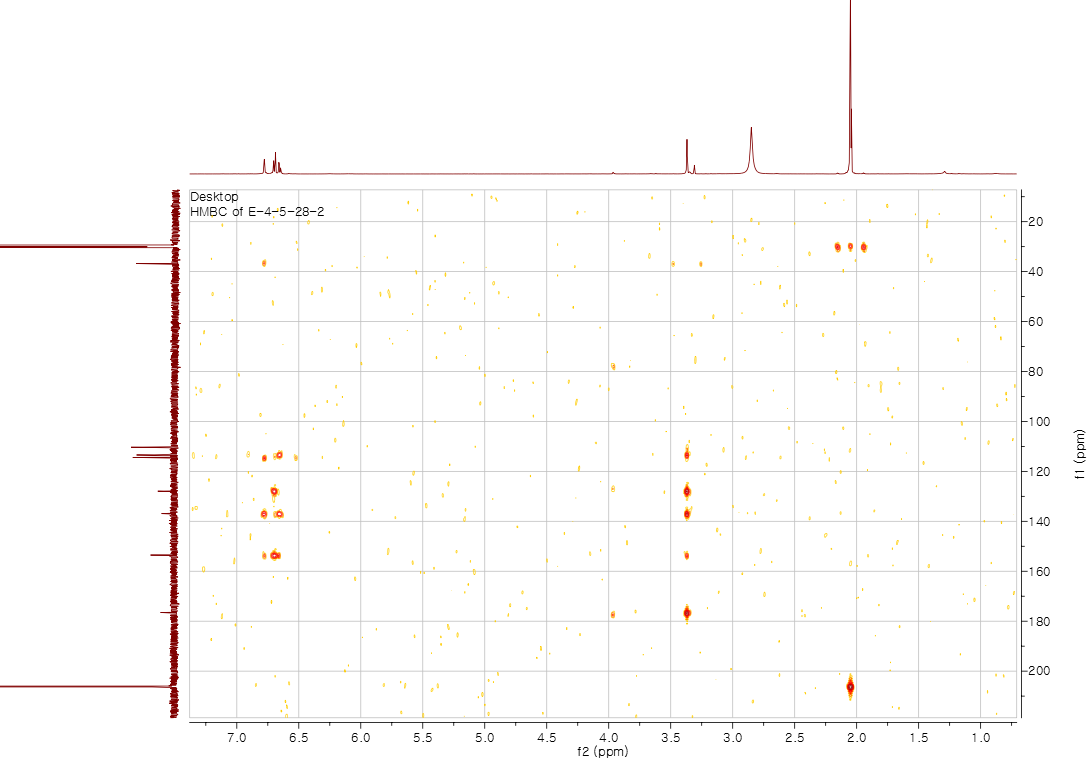


**Figure S7.** HMBC data of compound **1**

**Figure S8**. ^1^H NMR spectrum of compound **2** (600 MHz, D_2_O)

**Figure S9**. ^13^C NMR spectrum of compound **2** (150 MHz, D_2_O)

**
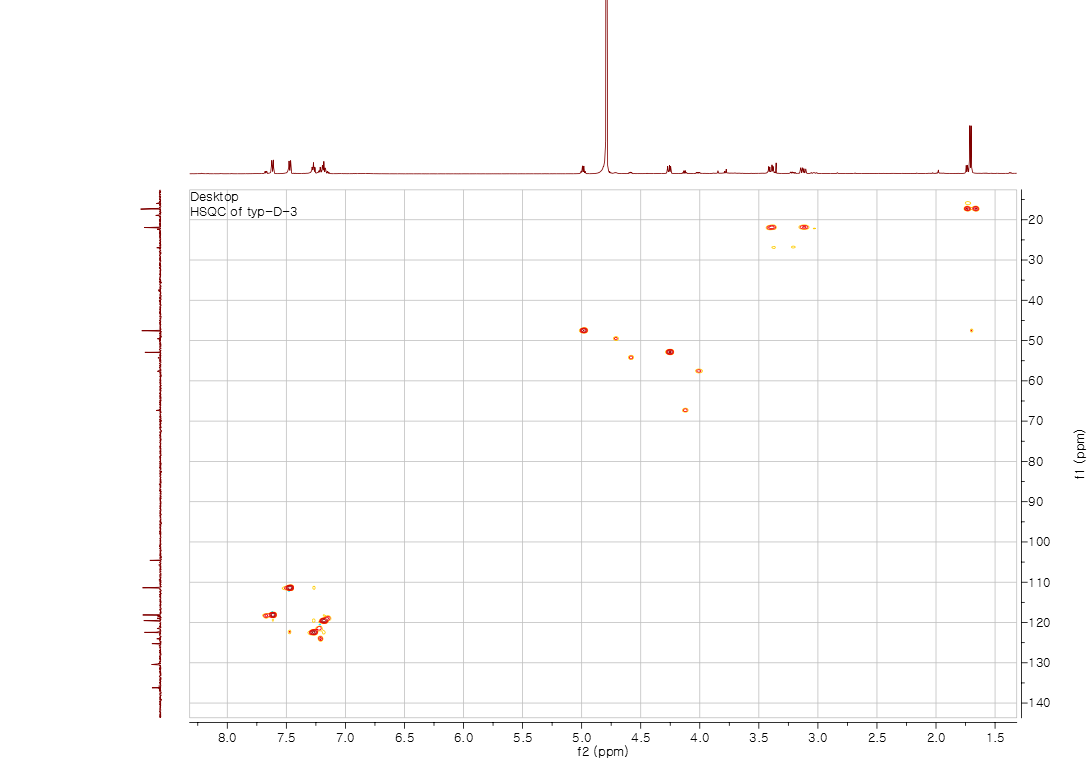
**

**Figure S10.** HSQC data of compound **2**

**
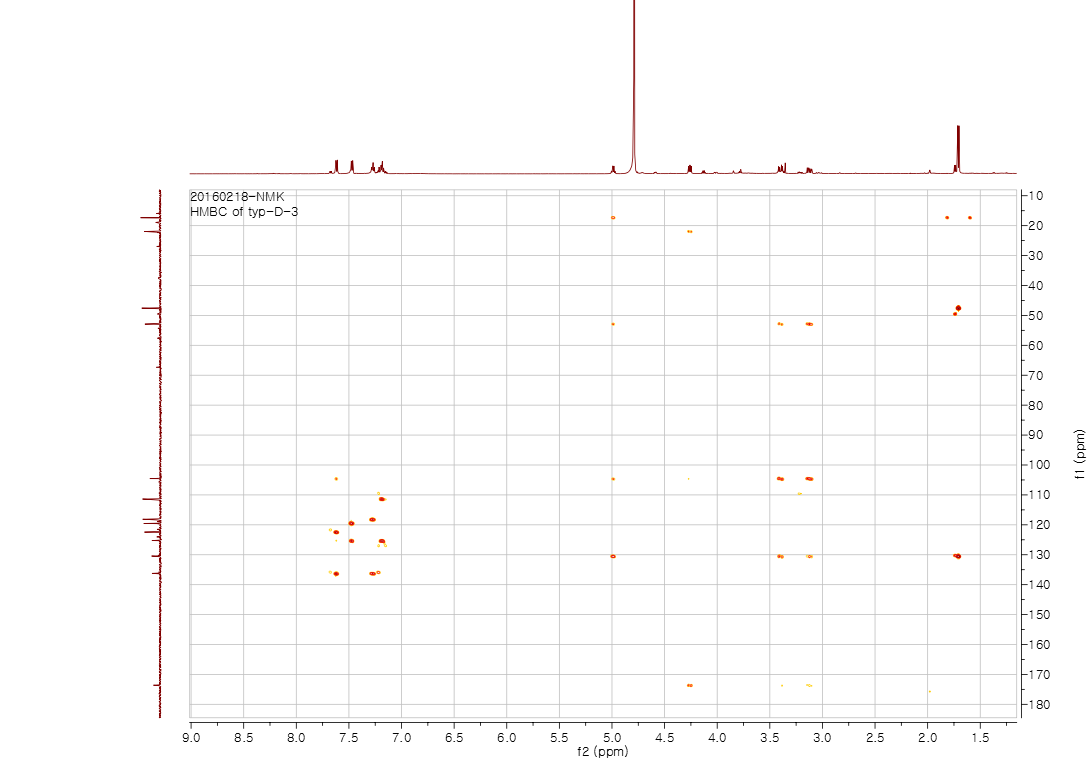
**

**Figure S11.** HMBC data of compound **2**

**Figure S12.** ^1^H NMR spectrum of compound **3** (600 MHz, DMSO-*d_6_*)

**Figure S13.** ^13^C NMR spectrum of compound **3** (150 MHz, DMSO-*d_6_*)

**Figure S14.** ^1^H NMR spectrum of compound **4** (600 MHz, DMSO-*d_6_*)

**Figure S15.** ^1^H NMR spectrum of compound **5** (600 MHz, DMSO-*d_6_*)

1. **Franke B, Akkerman JW, Bos JL.** Rapid Ca2+-mediated activation of Rap1 in human platelets. EMBO J. 1997 Jan 15;16(2):252-9.

2. **Lee W, Ku SK, Bae JS.** Antiplatelet, anticoagulant, and profibrinolytic activities of baicalin. Arch Pharm Res. 2015 May;38(5):893-903.

3. **Dixon M.** The determination of enzyme inhibitor constants. Biochem J. 1953 Aug;55(1):170-1.

4. **Izuhara Y, Takahashi S, Nangaku M, et al.** Inhibition of plasminogen activator inhibitor-1: its mechanism and effectiveness on coagulation and fibrosis. Arterioscler Thromb Vasc Biol. 2008 Apr;28(4):672-7.

5. **Goto S, Tamura N, Ishida H.** Ability of anti-glycoprotein IIb/IIIa agents to dissolve platelet thrombi formed on a collagen surface under blood flow conditions. J Am Coll Cardiol. 2004 Jul 21;44(2):316-23.

6. **Ku SK, Han MS, Lee MY, Lee YM, Bae JS.** Inhibitory effects of oroxylin A on endothelial protein C receptor shedding in vitro and in vivo. BMB Rep. 2014 Jun;47(6):336-41.

7. **Wu CC, Wu CI, Wang WY, Wu YC.** Low concentrations of resveratrol potentiate the antiplatelet effect of prostaglandins. Planta Med. 2007 May;73(5):439-43.

8. **Grynkiewicz G, Poenie M, Tsien RY.** A new generation of Ca2+ indicators with greatly improved fluorescence properties. J Biol Chem. 1985 Mar 25;260(6):3440-50.

9. **Bae JS.** Antithrombotic and profibrinolytic activities of phloroglucinol. Food Chem Toxicol. 2011 Jul;49(7):1572-7.

10. **Kim TH, Ku SK, Bae JS.** Antithrombotic and profibrinolytic activities of eckol and dieckol. J Cell Biochem. 2012 Sep;113(9):2877-83.
